# Drivers of Energy Consumption in Portuguese Hospitals - a panel analysis

**DOI:** 10.1093/eurpub/ckac131.164

**Published:** 2022-10-25

**Authors:** J Chen-Xu, V Moutinho

**Affiliations:** 1 NOVA National School of Public Health, Lisbon, Portugal; 2 Public Health Unit, Primary Health Care Cluster Baixo Mondego, Coimbra, Portugal; 3 Management and Economics Department, University of Beira Interior, Covilhã, Portugal

## Abstract

**Background:**

Buildings represent about 40% of global energy consumed, playing an important role in sustainable transformation. Health institutions account for 10% of the energy consumed by the commercial sector, however public policies do not cover energy management in this setting. Energy poverty in hospitals, such as poor heating, affect patients’ and workers’ health and wellbeing. As such, this study aims to analyse drivers of electricity and natural gas consumption of Portuguese hospitals.

**Methods:**

Data regarding 25 hospitals in Portugal’s National Health Service from 2015-2020 were analysed as logarithms in a panel of 18 trimesters, using Panel-corrected Standard Errors estimators. The PSAR model considers the presence of an autocorrelation within the panel, and the Hetonly modeling deals with heteroscedasticity and autocorrelation.

**Results:**

There is an elastic elasticity and a statistical significance of electricity and natural gas consumption in relation to the number of beds occupied, with a greater variation of natural gas than electricity consumption. In turn, there is a predominance of an inelastic elasticity of electricity and natural gas consumption in relation to the number of workers and number of emergency episodes.

**Conclusions:**

Energy consumption is highly influenced by the number of beds occupied, highlighting the need for a strategic management of energy and planning of hospital capacity. Overall, the positive absolute effects of the elasticities for energy consumption reveal that hospitals do not use effective measures aimed at rational and cleaner energy consumption, requiring a paradigm shift in the energy matrix. Increasing energy efficiency policies and environmental practices, such as autonomous energy production and renewable energies, might effectively contribute in reducing conventional energy consumption and associated energy poverty, which in turn improve patients’ and workers’ conditions within the hospital and their health and wellbeing.

**Key messages:**

• Strategic energy management and capacity planning can contribute to lower energy poverty and a paradigm shift in the energy matrix.

• Hospital sustainability policies must include energy management, including autonomous energy production and investment in renewable energies.

